# The influence of structural gradients in large pore organosilica materials on the capabilities for hosting cellular communities†

**DOI:** 10.1039/d0ra00927j

**Published:** 2020-05-05

**Authors:** Hannah Bronner, Anna-Katharina Holzer, Alexander Finke, Marius Kunkel, Andreas Marx, Marcel Leist, Sebastian Polarz

**Affiliations:** Department of Chemistry, University of Konstanz, Universitätsstraße 10 D-78457 Konstanz Germany; Department of Biology, University of Konstanz, Universitätsstraße 10 D-78457 Konstanz Germany; Institute of Inorganic Chemistry, Leibniz-University Hannover Callinstrasse 9 D-30167 Hannover Germany sebastian.polarz@aca.uni-hannover.de

## Abstract

Cells exist in the so-called extracellular matrix (ECM) in their native state, and numerous future applications require reliable and potent ECM-mimics. A perspective, which goes beyond ECM emulation, is the design of a host-material with features which are not accessible in the biological portfolio. Such a feature would, for instance, be the creation of a structural or chemical gradient, and to explore how this special property influences the biological processes. First, we wanted to test if macroporous organosilica materials with appropriate surface modification can act as a host for the implementation of human cells like HeLa or LUHMES. It was possible to use a commercially available polymeric foam as a scaffold and coat it with a thiophenol-containing organosilica layer, followed by biofunctionalization with biotin using click chemistry and the subsequent coupling of streptavidin–fibronectin to it. More importantly, deformation of the scaffold allowed the generation of a permanent structural gradient. In this work, we show that the structural gradient has a tremendous influence on the capability of the described material for the accommodation of living cells. The introduction of a bi-directional gradient enabled the establishment of a cellular community comprising different cell types in spatially distinct regions of the material. An interesting perspective is to study communication between cell types or to create cellular communities, which can never exist in a natural environment.

## Abstract 2

Chemical and structural gradients in biofunctionalized organosilica–polymer nanocomposites control cell adhesion properties and open perspectives for artificial cellular community systems.

## Graphical abstract

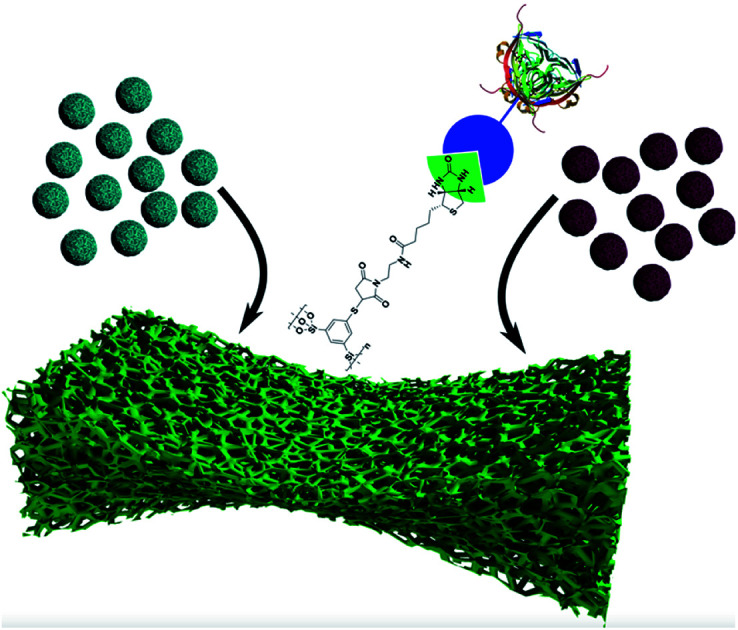

## Introduction

The size of matter used in contemporary nanoscience can be adjusted to the range of biological entities. As nanospecies are known to harm organisms their toxicologic aspects should always be considered.^1,2^ Any contact with the respective nanomaterial should be avoided, in case of toxicity. However, these materials present a powerful tool regarding biological applications. As research has accomplished a broad variety of chemical surface modifications, thus, an aim of current research has become the treatment of biological systems with nanomaterials on purpose. The emerging nanomedicine field is a good example,^3–5^ where materials are used for diagnostic or therapeutic purposes. The contact between organisms and the synthetic material is still intended to be short in duration for the vast majority of cases reported in the literature, like in nanomedicine. A tempting perspective is to design synthetic materials in such a way that they can be integrated with living cells in a more sustainable way, ultimately leading to persistent composites between living and synthetic matter. Such new materials may have unique properties and the synthetic constituent can be used to influence biological processes in an unprecedented way.

Cells in their natural environment are surrounded by the extracellular matrix (ECM), and huge effort was already undertaken trying to mimic it.^6,7^ To support cell adhesion, proliferation, and differentiation the materials must fulfil several requirements. Matrigel®, which is a combination of extracellular matrix proteins extracted from Engelbreth–Holm–Swarm tumours in mice, is a commonly used natural substrate for mammalian cell culture.^8^ One major drawback of tissue-derived substrates is their batch-to-batch variation and the presence of undesired impurities.^9^ Furthermore, it is difficult to equip Matrigel® or other soft-matter systems used for tissue engineering with a defined structure such as open porosity. Moreover, the majority of matrices known in the literature are structurally and chemically homogeneous over macroscopic dimensions. The latter feature is clearly distinct to the situation in real biological systems since living tissue is hierarchically organized. Consequently, the generation of synthetic and hierarchically structured materials is a challenge which could open up many new possibilities beginning with the creation of emulated organs and ending with cellular communities not existing in nature.

A less noted element of hierarchy is the occurrence of directionality in functional gradient materials.^10^ A sharp boundary is replaced by a transition from one feature to the next. The gradient can either be a structural one, for instance the change of pore-size over a certain a distance or characterized by a transition of chemical/surface properties. Thus, graded host materials offering different compartments for cells appear to be highly interesting. Before we report about our results going into this direction, information has to be given on, how to modify the surfaces of inorganic materials with groups making them biocompatible, how to structure the materials with special emphasis on gradient generation and what kind of gradient materials already exist in literature. Since, in nature there are plenty of materials that possess a functional gradient,^10–12^ it is desirable to establish porous biomaterials which imitate these properties.^13^ There is only limited literature on functionally graded biomaterials. Oh *et al.* have been able to synthesize a polycaprolactone (PCL) scaffold with gradually increasing pore size by a centrifugation method.^14^ They could show that different cell and tissue types have individualized pore size ranges for their effective growth. Sobral *et al.* used 3D plotting to establish a fibrous scaffold made from PCL and starch and found out that the type of gradient has an influence on the cell seeding efficiency.^15^ Cichocki *et al.* have already been using the versatility of PU foams in creating various porous networks.^16^ By thermal pre-treatment and application of pressure, they were able to give the foam a new form. They succeeded in creating porous alumina with different size gradients by infiltrating an aluminium oxide precursor and calcination of the foam. Even though this approach is a simple way to create every conceivable porous structure it has not been combined with a functional surface.

The simplest method for making a surface attractive for cell adhesion is physical adsorption of appropriate proteins. Next to proteins as laminin and vitronectin, fibronectin is part of the extracellular matrix (ECM) and crucial for the attachment of mammalian cells.^17^ The amino acid sequence RGD (Arg-Gly-Asp) is the entity in the protein which is responsible for cell adhesion.^17^ However, covalent attachment of the biomolecules is preferred,^18^ because desorption and leaching is avoided. Furthermore, undesired events such as conformational changes^19^ or incorrect orientation on the material thereby presenting an inactive site to the cell can be minimized.^20^

A valuable candidate as the synthetic constituent in the matrices is organosilica.^21,22^ Not only, there is a range of functional groups available for the attachment of biopolymers,^21,23^ it also has been proven to be non-toxic as indicated by multiple applications in a biological context.^24–27^ Organosilica can be structured in different ways from microporous, mesoporous to macroporous materials. The pore-size is a crucial factor regarding the possibility to host cells. So-called aerogels can be prepared by a sol–gel process,^28,29^ but their pore-size, though in the macroporous regime (>100 nm), is too small for cells. Others have used templates for the generation of hierarchically ordered macro-/mesoporous systems with inverse opal morphology.^30,31^ For instance, Zhou *et al.* were able to immobilize lipase which can catalyse the esterification of levulinic acid into alkyl levulinates.^32^ Nevertheless, these systems are still not able to function as a scaffold for cell growth since their pore system is limited in its size. A promising approach is to use a polymer foam as a template and infiltrate the precursor solution thereby creating a composite material of polymer and organosilica. Such materials have for instance been investigated for bone tissue engineering.^33,34^ Chen *et al.* were able to synthesize a glass which replicated the structure of the polymer foam by immersing the foam in a glass slurry and pyrolysis of the foam.^35^ By this method and the addition of a block copolymer as a co-template it is also possible to create hierarchical macroporous and mesoporous glass scaffolds.^36^ The reader interested more in bioactive glasses is referred to the review articles by Jones *et al.*^23^ or Rahaman *et al.*^33^

The methodologies for organic modification of silica materials is highly progressed and has been featured in numerous excellent review articles over the years.^22,37–39^ Because almost all of the work published has been concentrated on mesoporous materials, it seems the full potential of organosilica chemistry is not yet exploited for materials with other, and in particular larger pores like aerogels or foam-templated glasses. For instance, our group established an entire family of silsesquioxane sol–gel precursors and the corresponding materials.^40–50^ We were able to introduce functional groups into the organosilicate material like azides and thiols, which are capable to undergo further modifications by click-chemistry^51,52^ like the 1,3-dipolar Huisgen cyclo-addition or the thiol–ene reaction.^47,49,50^

**Scheme 1:**
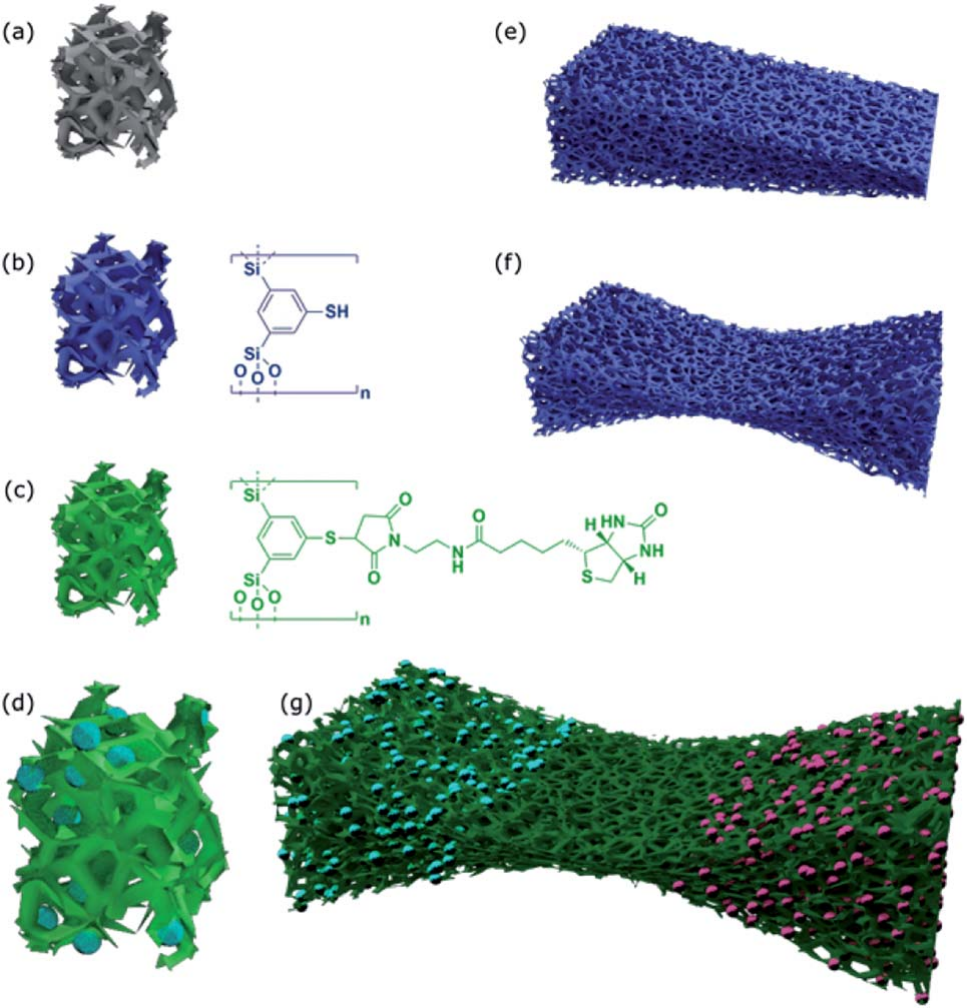
Overview about materials prepared in this study. (a) Polymer foam structure as a template. (b) Surface modification with thiophenol organosilica materials. (c) Attachment of a biotin moiety by click-chemistry. (d) Cells hosted by the material. (e) A material possessing a single structural gradient. (f) A bidirectional gradient material leading to an adjustable structural barrier. (g) Bidirectional gradient material as hosts for spatially separated co-culture of two human cell lines.

The first of our tasks is to apply the organosilica methodology developed in our group to prepare materials, with pores large enough to host living cells (Scheme 1). The next step in order to generate a material with biologically relevant surface properties by exploiting click-chemistry.^52^

However, we also want to go one step further, by creating a graded material and thereby creating a barrier for cell diffusion and develop a material which is a promising candidate for co-culture of cells.

## Materials and methods

### Materials

The synthesis that acquired inert gas atmosphere was performed using general Schlenk techniques under argon atmosphere. The solvents were dried according to the standard literature and stored under argon. All starting materials used for the synthesis were purchased from commercial sources unless stated differently.

### Synthetic procedures

#### Materials

All chemicals were received from Sigma-Aldrich, except d-biotin was purchased from IrisBiotech. The experiments were performed using a commercially available reticular polyurethane foam with PPI 50 (pores per inch); modulor soft foam article number 0333035. Other foams can be used as well shown for the BASF Basotect-W foam.

#### Organosilica precursor

**Scheme 2:**
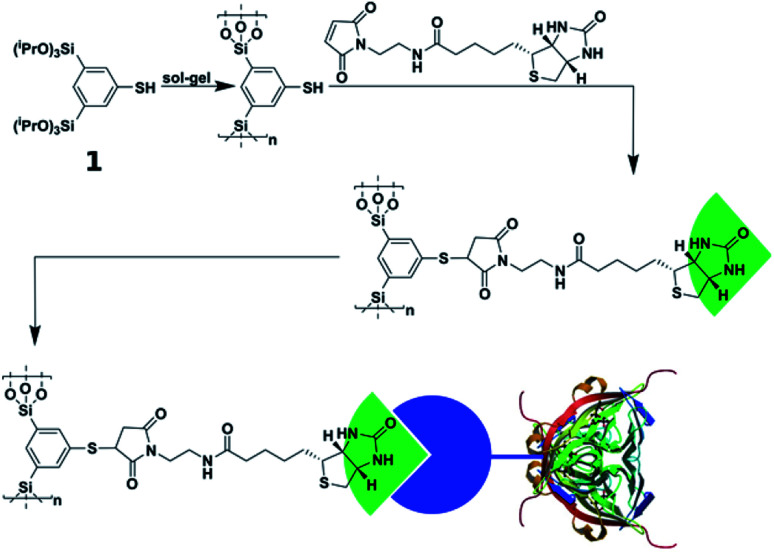
Synthesis steps of thiophenol organosilica (UKON-2j, 1) to biotin-modified and streptavidin–fibronectin modified surfaces (PDB ID 1MM9 (ref. 53)).

1,3-bis-(Triethoxysilyl)-5-thiophenol (UKON-2j, see Scheme 2 (1)) was prepared as reported in the literature.^47^ Further details are given in the ESI.†

#### Thiophenol organosilica foams

Prior to use, the commercially available PU foams are precleaned in boiling toluene, acetone and ethanol for 2 h to dissolve any remaining soluble components. The foams are washed extensively with ethanol and dried in the drying chamber. A typical preparation of PU–organosilica foams is as follows: a total of 0.52 g of the thiophenol–organosilane precursor (1) were dissolved in 3 mL of ethanol and 75 μL of 1 M HCl were added under stirring. The mixture was prehydrolyzed for 3 h at 60 °C. The pre-treated PU foam was cut into suitable pieces and the prehydrolyzed solution was infiltrated into the foam. To remove any air in the pores the foam was compressed while in solution. The material was aged for 1 d.

Biotin-maleimide was synthesized according to a previously reported process.^54^ Further details are given in the ESI.†

#### Click-modification of the organosilica surfaces

The PU-organosilica material was weighted before and after infiltration with the sol–gel precursor (1). The weight of the attached organosilica was calculated. 1 eq. of PU–organosilica material and 1 eq. of biotin-maleimide were dissolved in 4 mL of 0.1 M sodium phosphate buffer (pH = 8) and shaken for 24 h. The resulting material was washed with water for several times.

Ellman test was carried out according to literature.^55^

#### Fibronectin coating of the biotinylated surface

A fibronectin streptavidin fusion protein was prepared according to an established protocol.^56^ Biotinylated thiophenol organosilica foams were incubated with 10 μM fibronectin streptavidin fusion protein in PBS for 30 min at 37 °C followed by washing once with PBS and 3 times with cell culture medium.

#### Variation of the porosity

To modify the porous structure of the polyurethane foams the washed PU foam is cut into suitable pieces and compressed between two glass slides. To generate gradients, the materials are put into a mould with an angle according to the steepness of the gradient. The compressed foams are heated to 150 °C for 5 h. The heat treatment ensures that the foams retain their form even after removal of the glass slides or mould.

### Cell culture experiments

#### HeLa cell culture

HeLa cells were maintained in HeLa culture medium (DMEM, high glucose, GlutaMAX™ Supplement, pyruvate supplemented with 10% fetal calf serum and 1% penicillin/streptomycin) at 37 °C and 5% CO_2_. Cells were passaged every 3–4 days. To detach cells from the plate, cells were washed once with PBS and enzymatically dissociated with trypsin. Cells were washed off with culture medium. For maintenance, cells were reseeded in lower density and defined cell numbers were used for experiments.

#### LUHMES cell culture

tRFP-overexpressing LUHMES were created by infecting the LUHMES cell line with a lentivirus overexpressing tRFP according to Schildknecht *et al.*^57^ Cells were grown at 37 °C and 5% CO_2_ and maintained and differentiated according to previous protocols.^57–59^

Cytotoxicity assessment was performed using HeLa cells and standard Lactate dehydrogenase (LDH) release assay was performed. Further details are given in the ESI.†

#### Assessment of biocompatibility of organosilica foams

Onto each organosilica foam, 2 × 10^6^ HeLa cells were seeded in HeLa culture medium. After 17 h, viable cells were stained with Calcein-AM (1 μM) for 30 min. Imaging was performed at excitation wavelengths 350 nm and 488 nm.

### Co-culture in bidirectional structural gradient organosilica material

tRFP-overexpressing LUHMES were differentiated in LUHMES differentiation medium Advanced DMEM/F12, 1× N2 supplement, 2 mM l-glutamine, 1 mM dbcAMP (Sigma), 1 μg mL^−1^ tetracycline (Sigma) and 2 ng mL^−1^ recombinant human GDNF (R+D Systems). After 2 days, cells were enzymatically dissociated with trypsin, collected in Advanced DMEM/F12 and centrifuged at 300×*g* for 5 min at RT. The cell pellet was resuspended in differentiation medium and 2 × 10^6^ cells were seeded onto one side of biofunctionalized bidirectional structural gradient organosilica material.

18 h after seeding of LUHMES cells, HeLa cells that were pre-stained for 30 min with Calcein-AM (1 μM) were seeded on the other side of the organosilica material. After another 6 h, imaging was performed at excitation wavelengths 350 nm, 488 nm and 535 nm.

### Analytical methods

NMR-spectra were acquired on a Bruker Avance III 400 spectrometer using CDCl_3_ or DMSO-d_6_ as a solvent. The ESI-MS data were recorded using a Bruker micrOTOF II spectrometer. UV/VIS spectroscopic analysis was performed using an Agilent Cary 60 spectrometer. SEM measurements were performed using a Zeiss FESEM Auriga 40™ Crossbeam and a Hitachi TM3000 Tabletop SEM with a Bruker Quantax EDX Detector for the EDX measurements. EDX line scans were performed at a Zeiss Gemini 500 equipped with an Oxford EDX Ultim Max 100 detector. FT-IR spectra were recorded by using a PerkinElmer Spectrum 100 spectrometer using ATR unit. TGA measurements were measured on a Netzsch STA449 F3 Jupiter. All measurements were performed under oxygen atmosphere with 80 mL min^−1^ flowrate and a heating rate of 10 K min^−1^. Fluorescence microscopy was performed at labelled excitation wavelengths using a Zeiss Axio Observer Z1 microscope.

## Results and discussion

### Thiol-functionalized organosilica and aerogels as impractical host materials

Due to the high versatility of the thiol–ene click-chemistry, we focus on the organosilica system containing thiophenol as an organic constituent (see Scheme 1). Since aerogels possess a macroporous structure and high porosity,^60–62^ we hoped the pore-size can be made large enough to host cells. The preparation of the aerogels using 1,3-bis-(triethoxysilyl)-5-thiophenol^47,50^ as a sol–gel precursor was successful, so was the modification of the surfaces with biotin groups followed by streptavidin–fibronectin attachment (data are summarized in ESI Fig. S1;† see also Scheme 2). Comparison of the sizes of the pores (*D*_P_ ≈ 100–200 nm) according to scanning electron microscopy (SEM) micrographs with the size of HeLa cells (10–50 μm (ref. 63)) indicates the size of the voids is by a factor of ×100 too small for hosting the cell. As a consequence, cells can only sit on top of the material, but they cannot enter at all. As we saw no possibility to increase the pore-size of the aerogels that much and still have a mechanically stable material, the aerogel-approach was dropped immediately.

### Preparation of thiol functionalized organosilica coating of polymer foams and structural gradients

Given the arguments described in the previous paragraph about aerogel materials, our next approach was to use a material as a scaffold, whose surfaces can be coated with thiophenol-based organosilica. Polymer foams appeared as ideal candidates due to the following arguments. A large variety is commercially available with different chemical composition, different pore-sizes and three-dimensionally connected pore-systems. Many are toxicologically safe and, if intended, the polymer can be removed easily from the hybrid material. Last but not least, unlike inorganic solids as silica materials, polymer-foams have elastic mechanical properties.

**Fig. 1:**
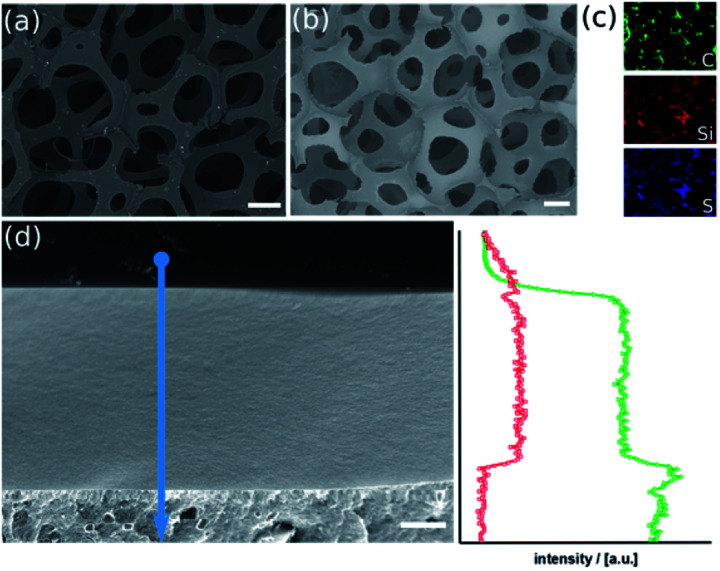
SEM micrographs of the PU-foam before ((a); scalebar = 200 μm) and after sol–gel coating with the thiophenol organosilica ((b); scalebar = 200 μm). Element-specific EDX-maps (c). SEM micrograph ((d), scalebar = 1 μm) of a cross-section of the organosilica layer (top) on the PU surface (bottom) and associated EDX line-scan (arrow); carbon trace (triangles, green) and silicon trace (squares, red).

The result of our experiments on commercially available poly-urethane (PU) foams are shown in Fig. 1.

In scanning electron microscopy, there are no structural differences between the materials before and after the sol–gel process visible (Fig. 1a and b). The material has maintained its porous character with inter-connecting pores in the range of several 100 μm and struts below 100 μm. Element-specific maps recorded by energy-dispersive X-ray spectroscopy, however, prove Si and S originating from the organosilica matrix are present. Element-specific mapping of pure PU foam only confirms the presence of carbon and oxygen (Fig. S2a and b†). This finding can be verified by elemental analysis (Fig. S2b†). The organosilica has formed a homogeneous and dense film on the polymer (Fig. 1d), which is clearly confirmed by EDX line-scan measurements. The latter conclusion is confirmed by infrared (IR) spectra, which contains the band at 1052 cm^−1^ characteristic for the Si–O–Si vibration and thermogravimetric (TGA) data shown in ESI Fig. S3.† Physisorption measurements cannot detect any surface area which confirms the density of the layer (Fig. S3c†).

The method described in the previous paragraph and in the experimental section can be applied to other polymers foams too, which allows to prepare the corresponding organosilica materials with different texture and pore-size. Two representative examples are given in ESI Fig. S4 and S5† and were analyzed by an analogous set of methods. The ability of the hydrolyzed thiophenol precursor to lead to homogeneous and dense coatings on polymer scaffolds is an advantage, but is not obvious. An important factor is, the hydrolysis and polycondensation reaction inside the polymer foams is done at acidic pH-values close to the isoelectric point of silica. Then, the formation of networks is preferred rather than particulate products (at basic conditions). Secondly, there has to be sufficient adhesion of the organosilica layer and the polymer. An attractive interaction between the two phases is promoted by the amphiphilic character of the hydrolyzed precursor. It can interact with hydrophilic surfaces *via* the silanol groups (SiOH) and also with hydrophobic surfaces due to the rather unpolar thiobenzene moiety.

**Fig. 2:**
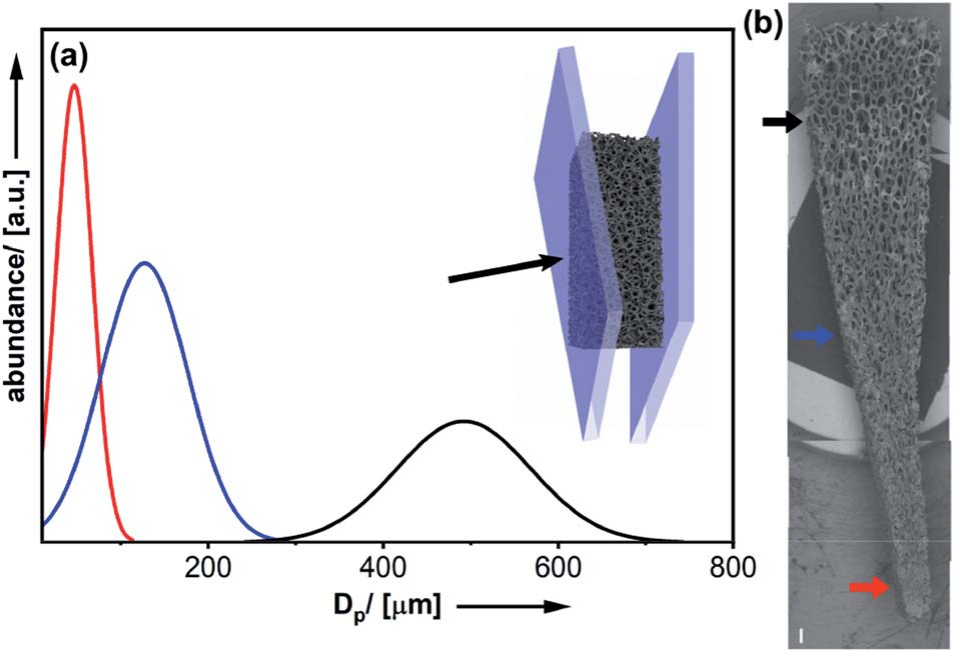
(a) Deformation of the polymer foam by mechanical force. Pore-sizes determined at three different positions from SEM micrographs ((b); scalebar = 500 μm).

As mentioned before, one advantage of using polymer foams as scaffolds is the possibility for deformation by mechanical force (see Fig. 2).^16^ Following deformation, the organosilica coating process can be performed. As a result, one obtains a material characterized by a persistent structural gradient with large pores on one side and almost closed pores at the other end.

**Fig. 3:**
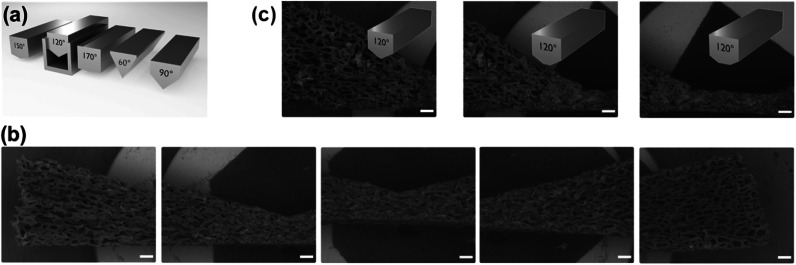
(a) Schematic deformation procedure leading to a bidirectional structural gradient organosilica material. SEM micrographs (scalebars = 500 μm) of a bidirectional 150° gradient (b) and variation of the extension of the pore-small region of a 120° bidirectional gradient (c).

Closer inspection of the SEM images (Fig. 2b) and comparison to the homogeneous material (Fig. 1b) also demonstrates, the shape of the pores has become anisotropic. Obviously, the structural gradient can be varied easily by adjusting the angle between the two plates (Fig. 2a). It was also possible to prepare materials with a bidirectional gradient. The foam is confined and deformed by a stamp, which defines the gradient angle (see Fig. 3a and S6†). Coating with thiophenol organosilica is performed as described before. The lower the angle of the kink in the stamp, the steeper is the gradient in the final material (Fig. 3b and c). By further modification of the stamp, it is also possible to change the extension of the zone characterized by small pores (Fig. 3c).

### Click-modification and biofunctionalization

**Fig. 4:**
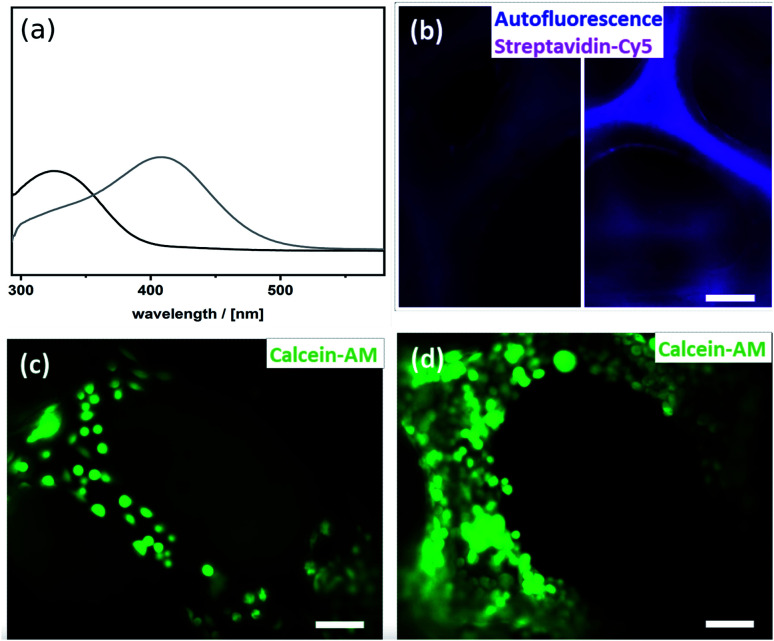
(a) Ellman test of the thiophenol organosilica foam before (grey) and after biofuntionalization of the surface (black). (b) Cy5-fluorescence (magenta) and autofluorescence (blue) of thiophenol organosilica foam incubated with Cy5-tagged streptavidin before (left) and after (right) biofunctionalization of the surface. HeLa cells (stained by calcein-AM) in the thiophenol organosilica foam (c) before and (d) after biofunctionalization of the surfaces; scalebar = 100 μm.

To utilize the material as a three-dimensional scaffold for cell growth its biocompatibility has to be confirmed. Therefore, we checked whether any soluble and potentially cytotoxic substances remained in the organosilica foam. For this, cell culture medium was used to produce eluates from the material, and we found that such media allowed similar growth and viability of HeLa cells as control medium. This finding confirms that no harmful substances were washed out of the material (Fig. S7†). As cells attached poorly to plain thiophenol organosilica foams (Fig. 4c), we aimed at the ECM mimicry of this three-dimensional scaffold to enhance cell attachment. To achieve this, it is necessary to coat the surface with protein epitopes known to promote cell adhesion. Consequently, the next step involved the modification of the thiol-groups by click-chemistry (Scheme 2). The pairing between biotin and streptavidin is a well-established tool in biological chemistry for the attachment of proteins.^64^ Therefore, we aimed to modify the organosilica surface using biotin-maleimide first, followed by the attachment of a fibronectin streptavidin fusion protein^56^ of which the firbronectin moiety confers binding to integrins like αvβ3 of HeLa cells.^65^ The procedure can be applied to all organosilica materials but is discussed here for the non-graded material shown in Fig. 1 as a proof-of-concept.

The post-functionalization reaction of the organosilica surface with biotin decreases the chemically accessible thiol groups. The number of remaining free thiols can be probed by the so-called Ellman test. The test is based on the reaction of 5,5′-dithiobis(2-nitrobenzoic acid) (DTNB, absorption at 325 nm) with a free thiol in the material and thus cleaving its disulfide bond to give 2-nitro-5-thiobenzoate (TNB), which has an absorption at 412 nm.^55^ Results from the Ellman test showed that the substantial number of reactive thiol groups prior to the post-functionalization reaction was reduced to nearly undetectable levels (Fig. 4a). In addition, one can use streptavidin conjugated to the fluorescent dye Cy5 as an indicator for biotinylation. The Cy5-fluorescence gives information on both the extent and distribution of biotinylation of the organosilica foam. As shown in Fig. 4b, an even distribution of biotin over the whole organosilica surface was confirmed. In a next step, the biotinylated organosilica material was incubated with a fibronectin streptavidin fusion protein. To prove that biofunctionalization of the surface was successful, HeLa cells were seeded onto the material. Attachment of the cells to the organosilica foam was evaluated by calcein-AM staining which visualized the cells and indicated their viability. The live stain revealed complete covering of the biofunctionalized organosilica scaffold with HeLa cells (Fig. 4d), which is significantly different from cell growth observed on non-biofunctionalized organosilica materials (Fig. 4c). The drastically enhanced coverage of the material with live cells confirms successful biofunctionalization of the organosilica foam and its suitability for biological applications.

### Gradient materials as hosts for human cells

After having successfully proven the significantly higher settlement of HeLa cells on fibronectin-functionalized organosilica materials, the graded materials were tested next. First, the materials were post-functionalized in the same way as the non-graded materials. The successful reaction was visualized again with the Cy5 assay (Fig. S8†).

**Fig. 5:**
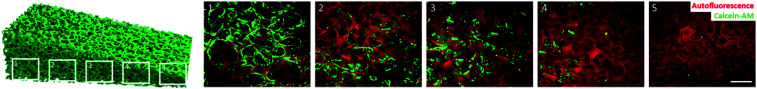
HeLa cell attachment to biofunctionalized structural gradient organosilica materials. Spreading of HeLa cells in the poresize gradient organosilica foams was analyzed 24 h after seeding. Calcein-AM positive and therefore viable HeLa cells (green) were found to populate areas with bigger pore sizes (1, 2 and 3). Areas exhibiting small pore sizes and high foam density were not populated with HeLa cells (4 and 5). Autofluorescence of the organosilica material (red) visualizes the gradient in pore size. Scalebar = 500 μm.

The decreasing pore size and increasing foam density is shown by the auto-fluorescence of the material itself (Fig. 5, red pseudo-colored). Seeding of HeLa cells depicted a considerable contrast in the different compartments of the material. The cells were analyzed 24 h after seeding onto the material. Staining with calcein-AM (green) confirmed the viability of the cells. A maximum of cells was found in the parts with normal sized pores (Fig. 5-1–3). The smaller the pores get, the fewer cells were found to attach (Fig. 5-4) until there were no more cells present in the material (Fig. 5-5). This effect can be explained by an increase of foam density, decrease of pore-size, and eventually a changed hydrophobic-hydrophilic balance of the material.

### Bidirectional gradient materials as hosts for spatially separated co-culture of two human cell lines

**Fig. 6:**
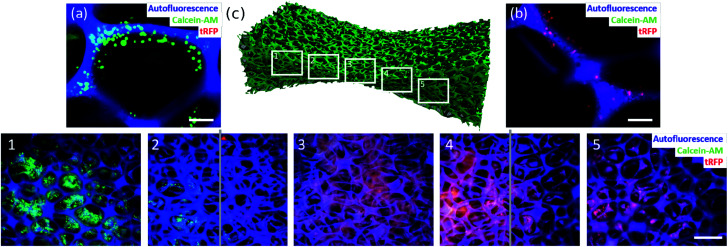
Spatially separated, indirect co-culture of two human cell lines in biofunctionalized organosilica materials achieved by bidirectional structural gradient. Calcein-AM positive HeLa cells (green) (a; scalebar = 100 μm) are exclusively found on the left side of the biofunctionalized organosilica material (c1 and c2), whereas tRFP-overexpressing LUHMES cells (red) (b; scalebar = 100 μm) were found solely on the right side of the material (c4 and c5). The barrier, indicated by the two grey lines, consisting of dense, small sized pores proved to be free of cells (detected fluorescence complies with material autofluorescence) (c): scalebar = 500 μm.

The design of materials mimicking the human body more closely is one major challenge in the design of multifunctional materials. The challenging aspect here is the culture of two or more cell types spatially separated from each other, however, keeping them indirectly connected *via* a shared culture medium. The shared culture medium allows the exchange of signaling molecules or metabolites between the different cell types. Such a system could be used as a model for single organs that are connected *via* body fluids. To tackle this challenge, we made use of the findings about cell behavior in graded materials. In the previous section we demonstrated that it is possible to build a material with a cell-free part. In a final experiment, this result was used to culture two human cell types spatially separated in one material. As a model we used HeLa and LUHMES cells. By designing a material with a bidirectional gradient (Fig. 6c), the small sized pores in the middle of the material built a barrier for diffusion due to their hydrophobic character. This barrier enabled the cultivation of HeLa cells exclusively on one side of the material (Fig. 6c-1 and c-2) and LUHMES cells spatially separated on the other side (Fig. 6c-4 and c-5). The barrier, depicted by the grey lines, remained free of cells (Fig. 6c-2–4).

## Conclusions

In the present work, we report the first synthesis of giant porous organosilica materials as a 3D scaffold for cell culture. We used commercially available PU foams as a structural template. Infiltration of the PU foam with a thiol containing benzene-bridged alkoxy silane precursor followed by sol–gel process allowed the coating of a PU foam with a functional layer organosilica layer. Since living tissue is hierarchically organized, one more challenging goal was to develop a material which displays a structural gradient. By inserting the PU foams into an appropriate mould, it was possible to create a material with any conceivable gradient. Therefore, it was possible to significantly decrease the pore diameter and create anisotropic pores. In order to make the surface biocompatible, thiol–ene chemistry was applied and a biotin-containing linker molecule was quantitatively attached to the surface. By exploiting the strong binding between biotin and streptavidin, the material was covered with a fibronectin–streptavidin fusion protein. Fibronectin is part of the ECM and responsible for cell adhesion. Comparisons of pure thiol–organosilica with fibronectin-functionalized surfaces confirmed the biocompatibility of the advanced material. One demanding task in the development of biomaterials is the urge to produce materials which represent more human *in vivo* like models. In doing so, one aspect is the co-cultivation of more than one cell type. By generating a pore size gradient, it was possible to establish a cell-free domain in the area of the drastically reduced pore size. In a final experiment we were able to establish a bidirectional gradient material with small pores in the middle. The barrier did not allow any cell diffusion into the other compartment and made it possible to co-culture two human cell types with a spatial separation in one material. Having different cell types cultured in one three-dimensional material makes it possible to investigate communication of cells that are not in direct contact with each other but are still capable of exchanging information *via* signalling molecules. This feature is accompanied by the technical advantage of easy handling as only one material has to be handled by the operator. Furthermore, large cell numbers can be cultured in small volumes on these three-dimensional porous materials, thereby also modelling hypoxia conditions of human tissue better.

## Conflicts of interest

There are no conflicts to declare.

## Supplementary Material

RA-010-D0RA00927J-s001
