# Supplementary material for: The influence of structural gradients in large pore organosilica materials on the capabilities for hosting cellular communities
Source: RSC Adv. 2020 May 5;10(29):17327–35. doi: 10.1039/d0ra00927j (PMC9053637; doi:10.1039/d0ra00927j)
Supplement: RA-010-D0RA00927J-s001 [file RA-010-D0RA00927J-s001.pdf]

Electronic Supplementary Material (ESI) for RSC Advances.
This journal is © The Royal Society of Chemistry 2020

The Influence of Structural Gradients in Large Pore Organosilica Materials on the Capabilities for Hosting Cellular Communities

Hannah Bronner, Anna-Katharina Holzer, Alexander Finke, Marius Kunkel, Andreas Marx, Marcel Leist and Sebastian Polarz

ELECTRONIC SUPPLEMENTARY INFORMATION

Part A - Material synthesis and characterization

Figure S1. Thiophenol-based aerogel materials.

(a) Synthesis route: Thiol-containing aerogel (UKON-2j aerogel).

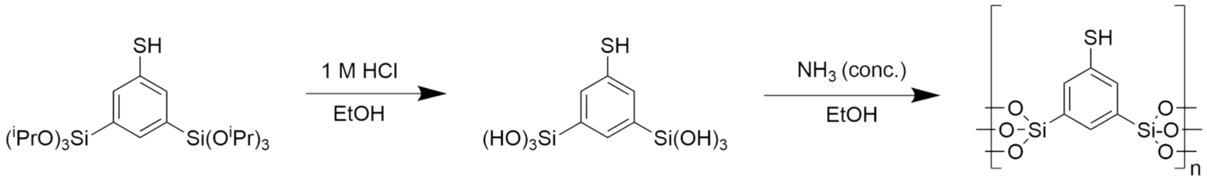

In a typical synthesis 0.22 mmol (115 mg, 1 eq.) of 1,3-bis-(triethoxysilyl)-5-thiophenol (UKON-2j) are dissolved in 2 mL of ethanol at room temperature. 50 µL of a 1 M hydrochloric acid solution are added under vigorous stirring. After hydrolyzing the solution for 1 h, 50 µL of concentrated ammonia was added and the solution is filled into a syringe and gelled overnight. The gelled monolith is removed from the syringe and immersed in acetone for three days. Thereby the acetone is replaced daily. To remain its porous structure for further analysis (FTIR, SEM, TGA, physisorption measurements) the monolith is dried supercritically. Monoliths which are post-functionalized with the biotin linker are not dried, but the acetone is exchanged with *N,N*-dimethylformamide over three days.

(b) Synthesis route: Biotin modification of the thiol-containing aerogel.

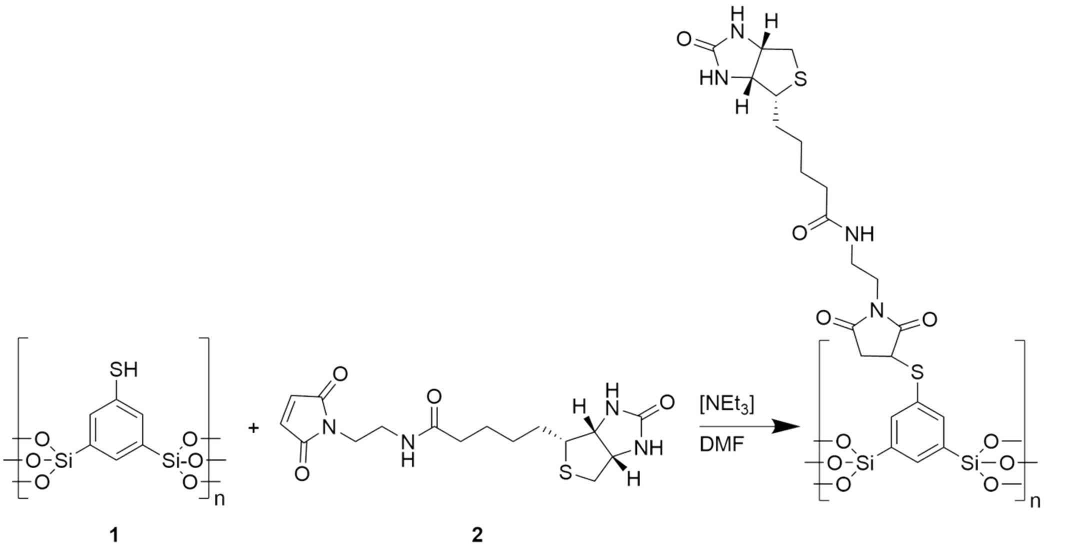

In a typical synthesis 0.022 mmol (8 mg, 1 eq.) MalBiotin (2) are dissolved in 5 mL of *N,N*-dimethylformamide at room temperature. 0.022 mmol (5 mg, 1eq.) of UKON-2j aerogel (1) which have been soaked with *N,N*-dimethylformamide are added to the reaction mixture. 0.21 mmol (30 µL, 9.5 eq.) of triethylamine are added and the material is shaken for 2 d. The post-functionalized material is washed with *N,N*-dimethylformamide and ethanol by immersing the monolith in the solvents for 3 days with daily exchange.

(c) SEM micrograph of the supercritically dried thiol-containing aerogel.

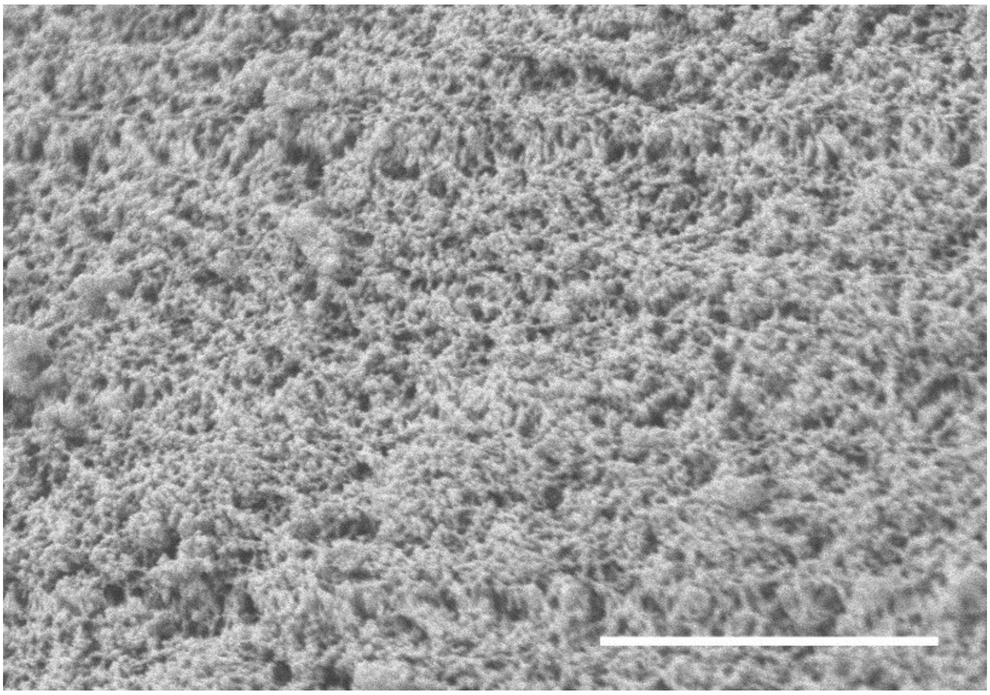

Scale bar = 2 µm.

(d) TGA-analysis.

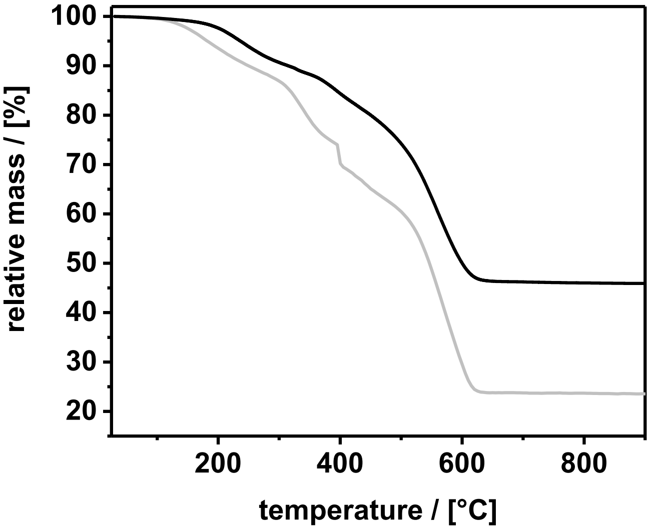

Black = UKON-2j aerogel; grey = UKON-2j aerogel post-functionalized with MalBiotin

Calculations yield in a post-functionalization degree of 83 %.

(e) FTIR-analysis.

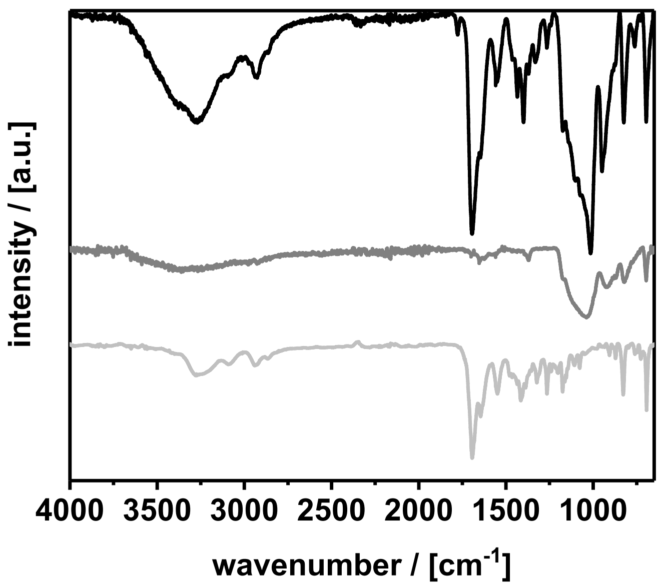

Black = UKON-2j aerogel post-functionalized with MalBiotin, dark grey = UKON-2j aerogel; light grey = molecular MalBiotin

The FTIR spectra of the UKON-2j aerogel post-functionalized with MalBiotin shows several significant vibration bands (black spectrum). The NH-vibration at ν = 3275 cm^{-1} and the CO-vibration at ν = 1693 cm^{-1}. These two vibrations in the composite material result from the clicked MalBiotin (compared to the identical signals in the light grey spectrum of pure MalBiotin). The Si-O-vibration at ν = 1020 cm^{-1} results from the UKON-2j silica vibration (compared to the identical signals in the dark grey spectrum of pure UKON-2j aerogels).

(f) Application of the post-modified UKON-2j aerogel as a host material for living cells.

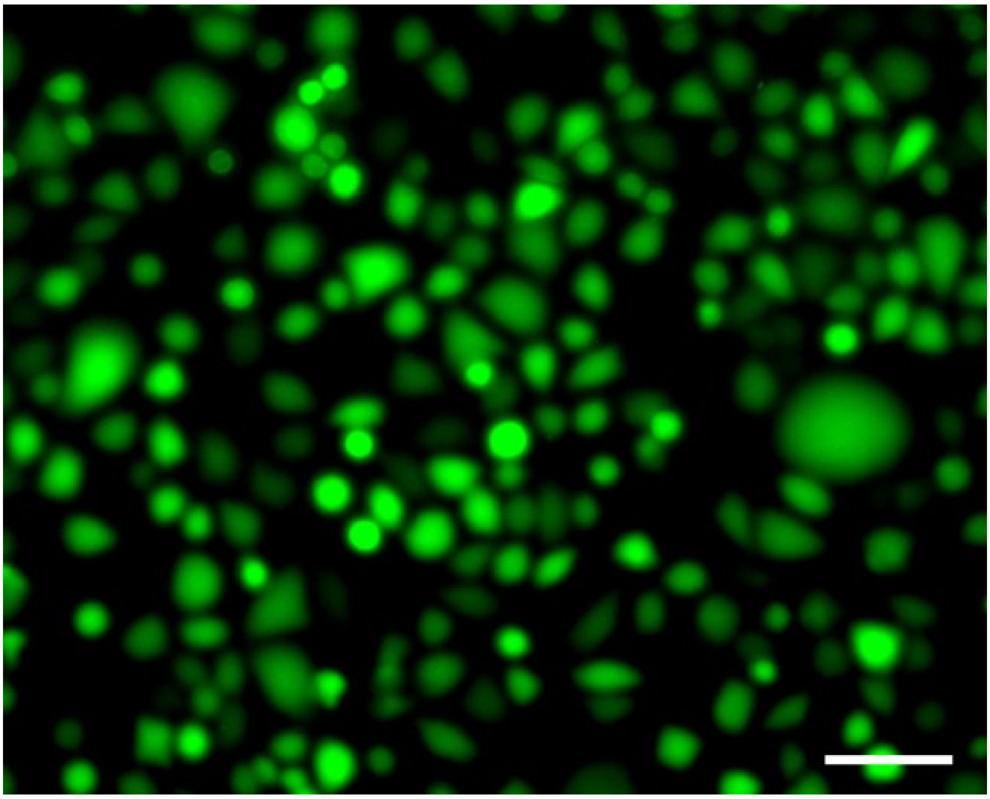

The UKON-2j aerogels which have been modified with MalBiotin are coated with the fusion protein streptavidin-fibronectin. The experimental procedure of coating with streptavidin-fibronectin as well as cell seeding correspond with the procedure for organosilica foams. The picture shows the cell growth of viable HeLa cells stained with calcein-AM (green) on biocompatible organosilica aerogel materials (surfaces modified by thiol-Biotin-Strep-Fib). Scale bar = 100µm.

Figure S2. Analysis of uncoated PU foam.

(a) SEM micrograph.

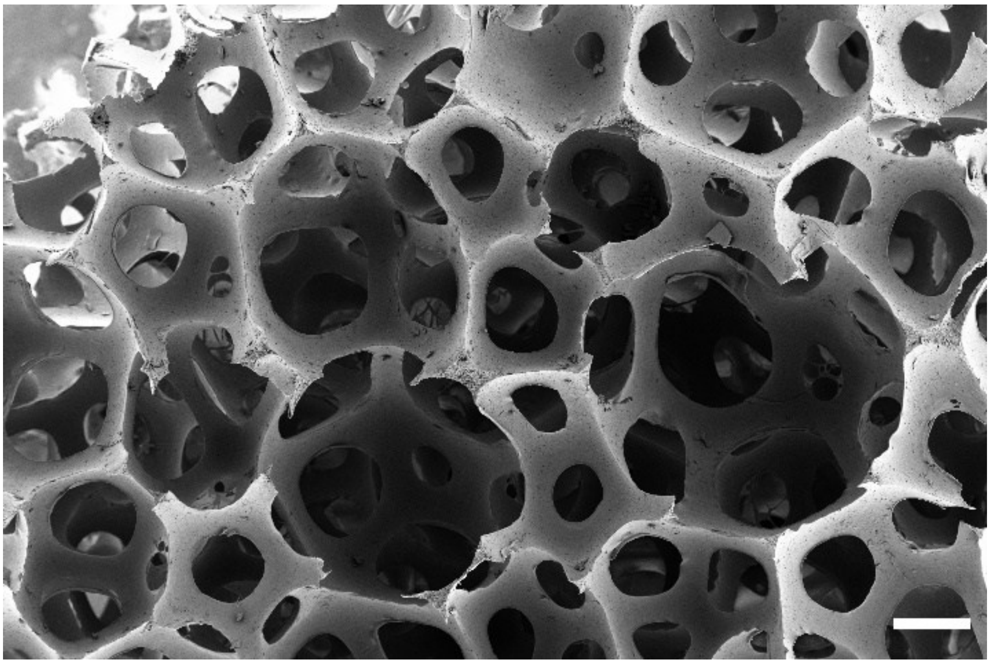

Scale bar = 200 µm.

(b) EDX analysis.

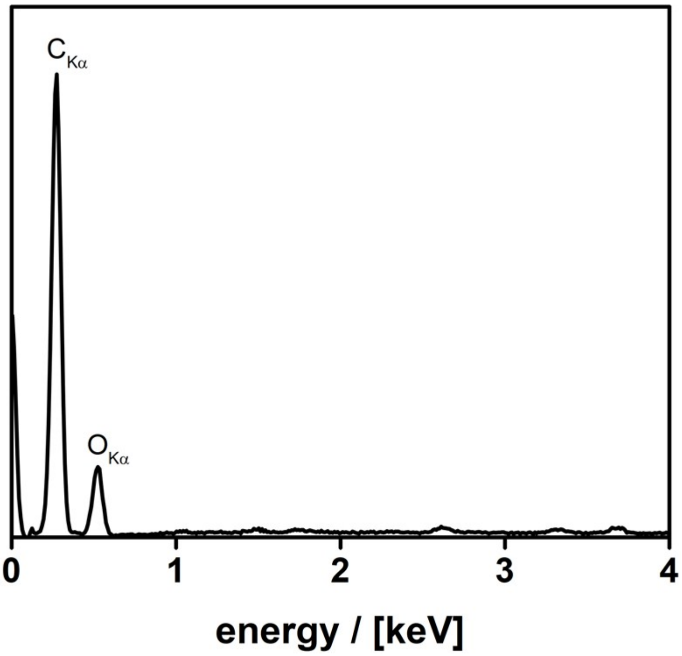

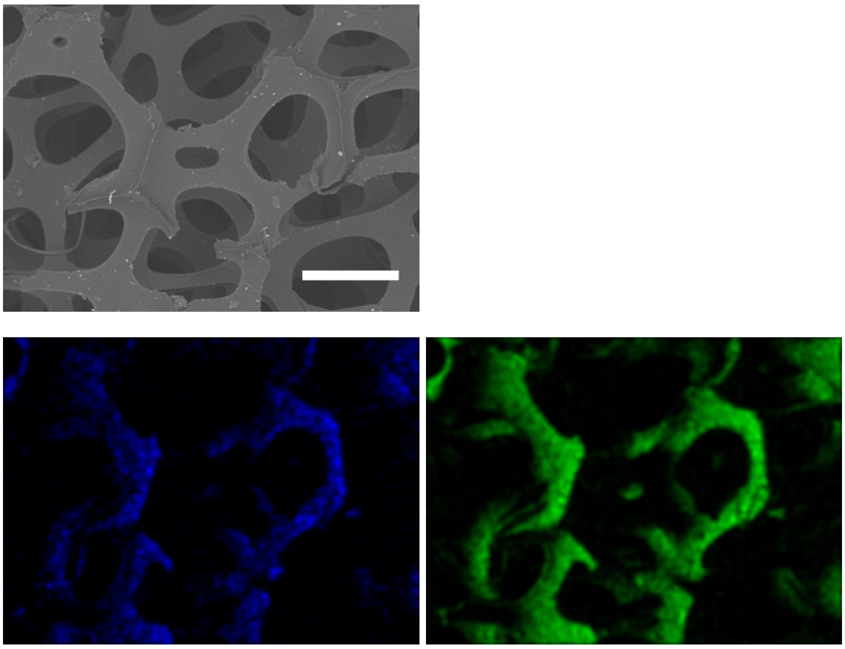

Scale bar = 500 µm.

Green: carbon, blue: oxygen.

(c) CHNS analysis.

N	4.625 %
C	61.54 %
H	7.815 %

Figure S3. Thiophenol-based organosilica PU foam composite.

(a) FTIR spectroscopy

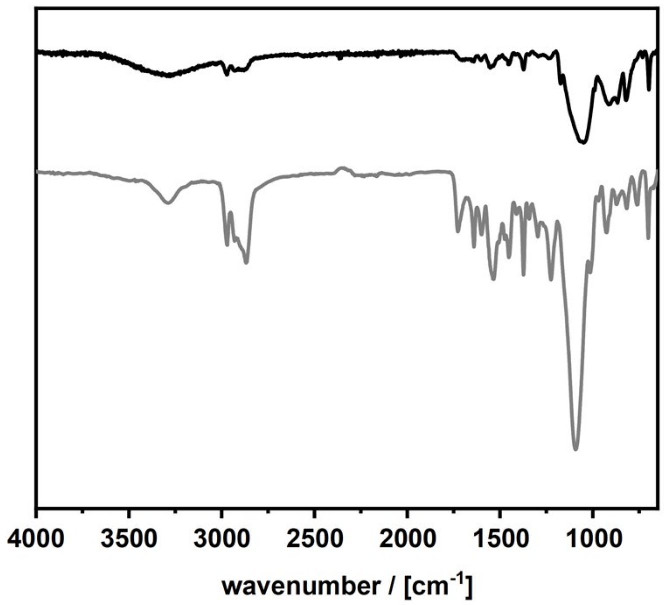

Black = Thiophenol-silica PU composite; grey = pure PU foam as a reference.

The strong Si-O stretching vibration is visible at 1052 cm^{-1} which can be clearly assigned to the organosilica part of the hybrid material. The signals for the aliphatic C-H vibrations at 2986 – 2847 cm^{-1} and the C=O vibration at 1717 cm^{-1} which result from the PU foam are still visible in the spectrum of the hybrid foam.

(b) TGA.

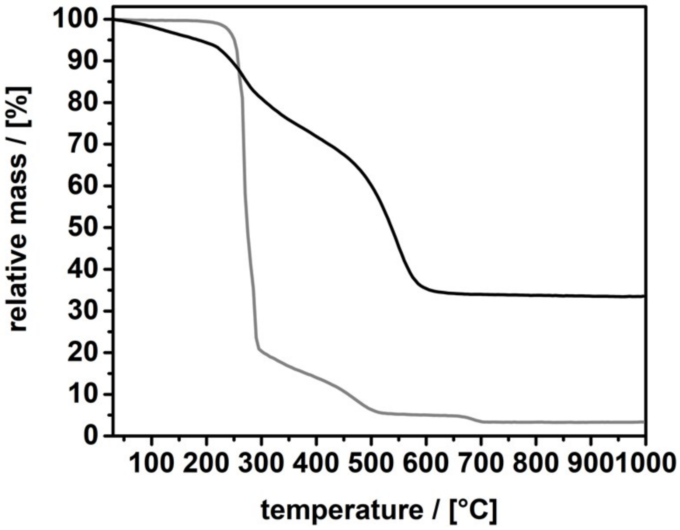

Black = Thiophenol-silica PU composite; grey = pure PU foam as a reference.

3 % of the total mass remains for the pure PU foam, the composite material has a remaining mass of 33 %. Under the assumption that the UKON-2j decomposes completely to SiO_2, a mass ratio of PU foam to UKON-2j of approximately 1:1 can be calculated.

(c) N_2 Physisorption.

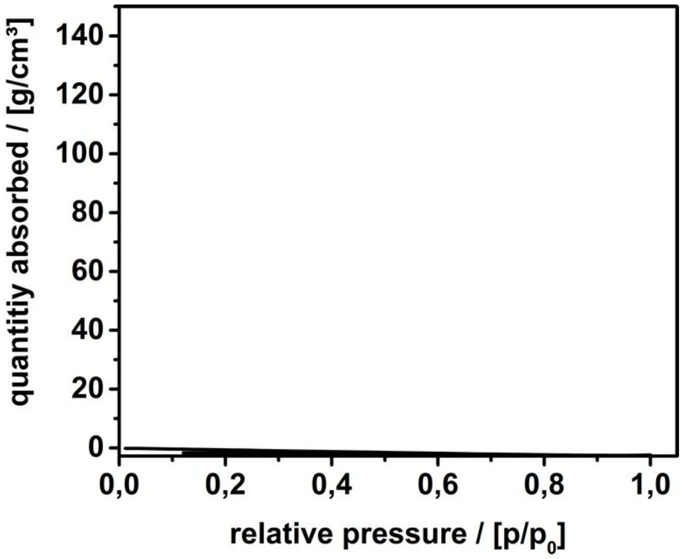

Black = Thiophenol-silica PU composite.

Figure S4. Thiophenol-based organosilica PU2 foam composite.

(a) SEM micrographs

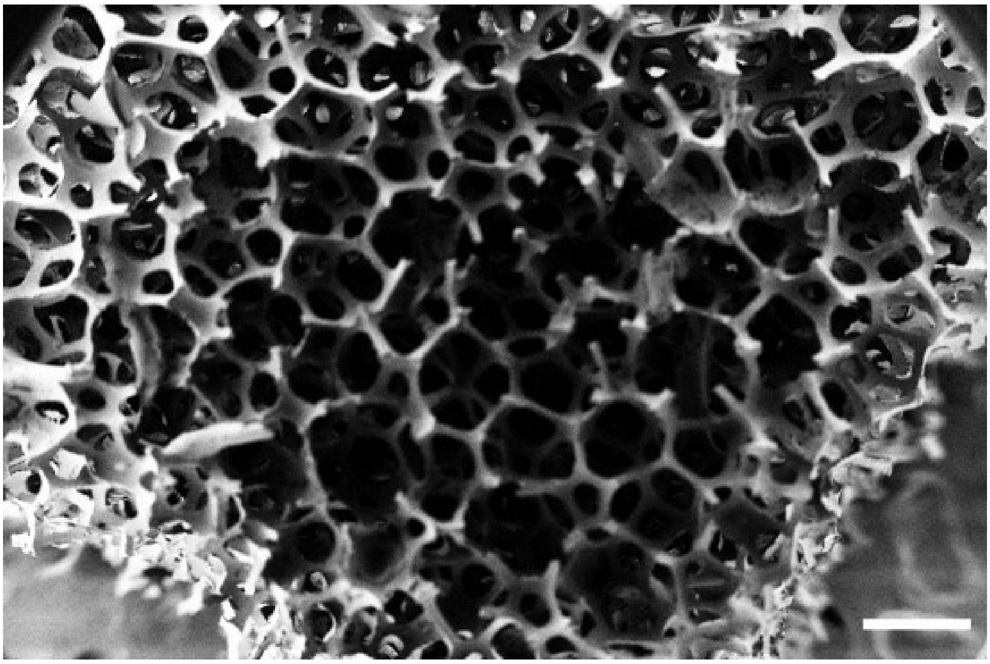

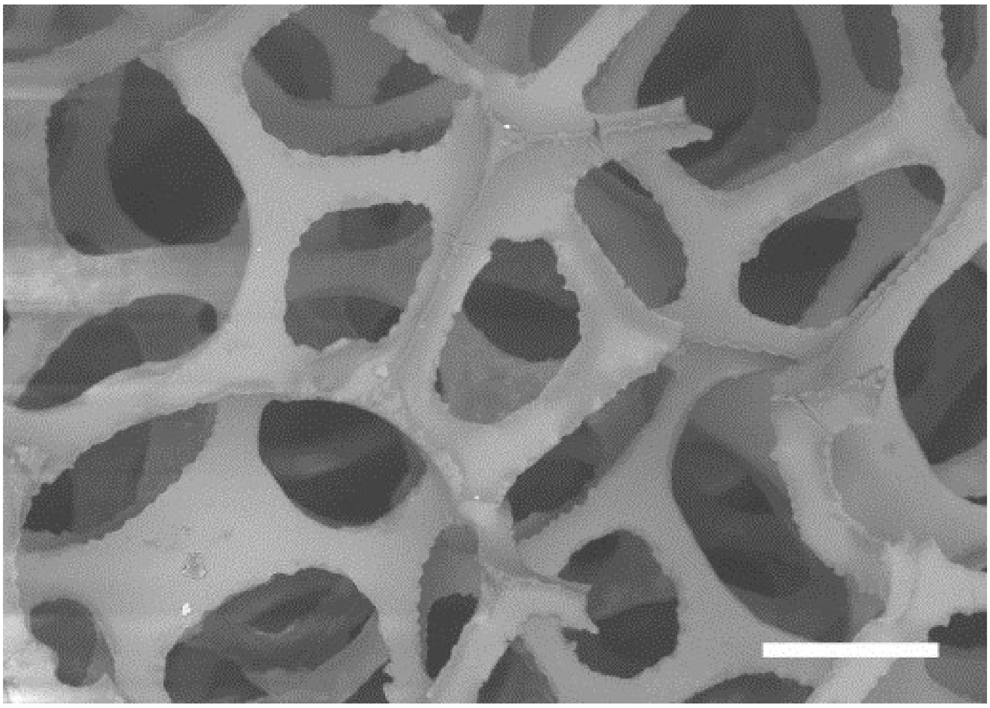

Scale bar = 500 μm.

(b) EDX analysis.

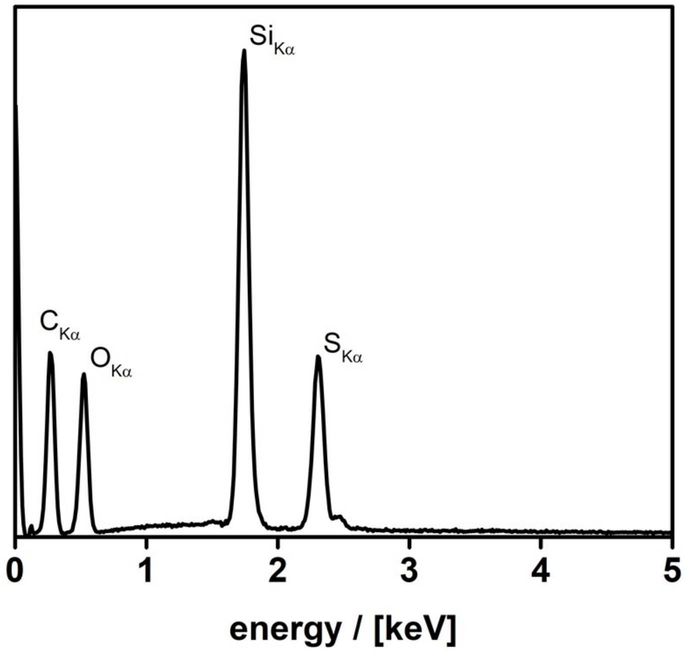

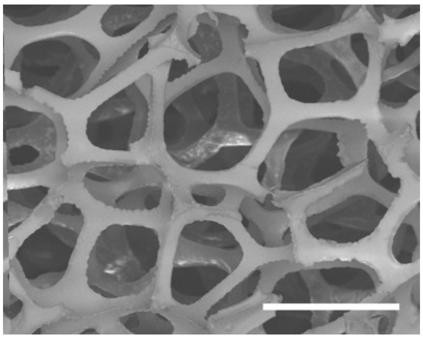

Scale bar = 200 µm.

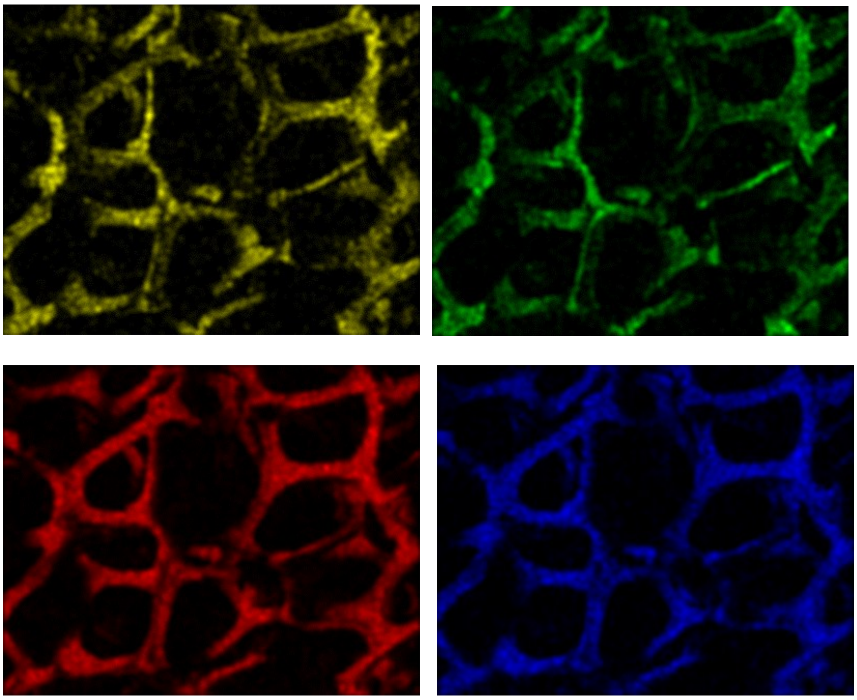

Green: carbon, Yellow: oxygen, Red: silicon, Blue: sulfur

Figure S5. Thiophenol-based organosilica Basotect® foam composite.

(a) SEM micrographs

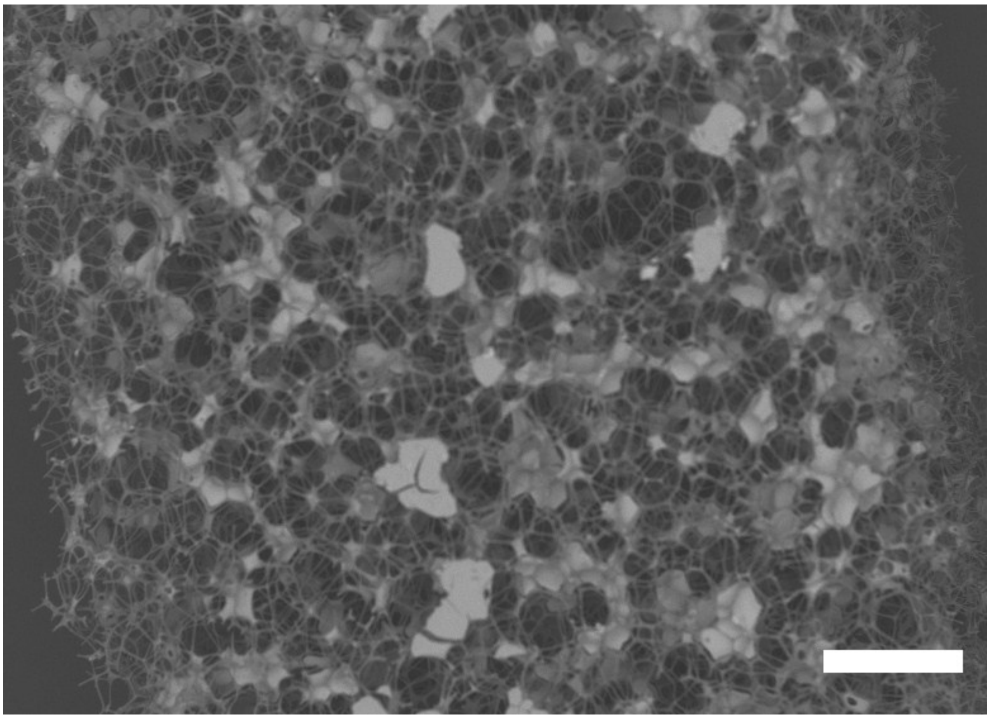

Scale bar = 500 µm.

(b) EDX analysis

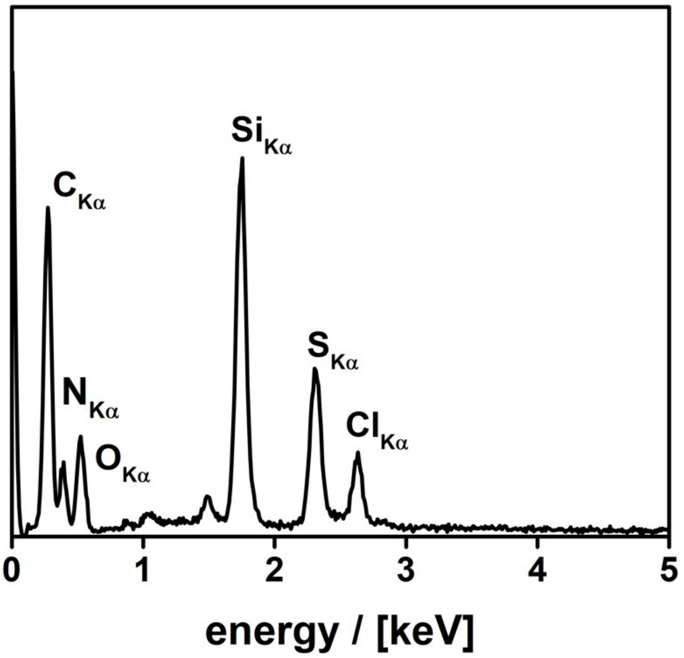

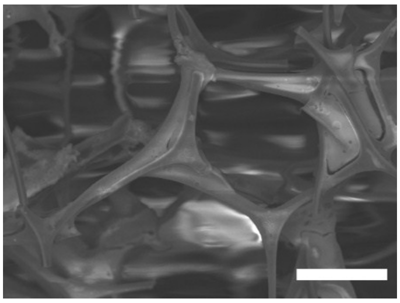

Scale bar = 50µm.

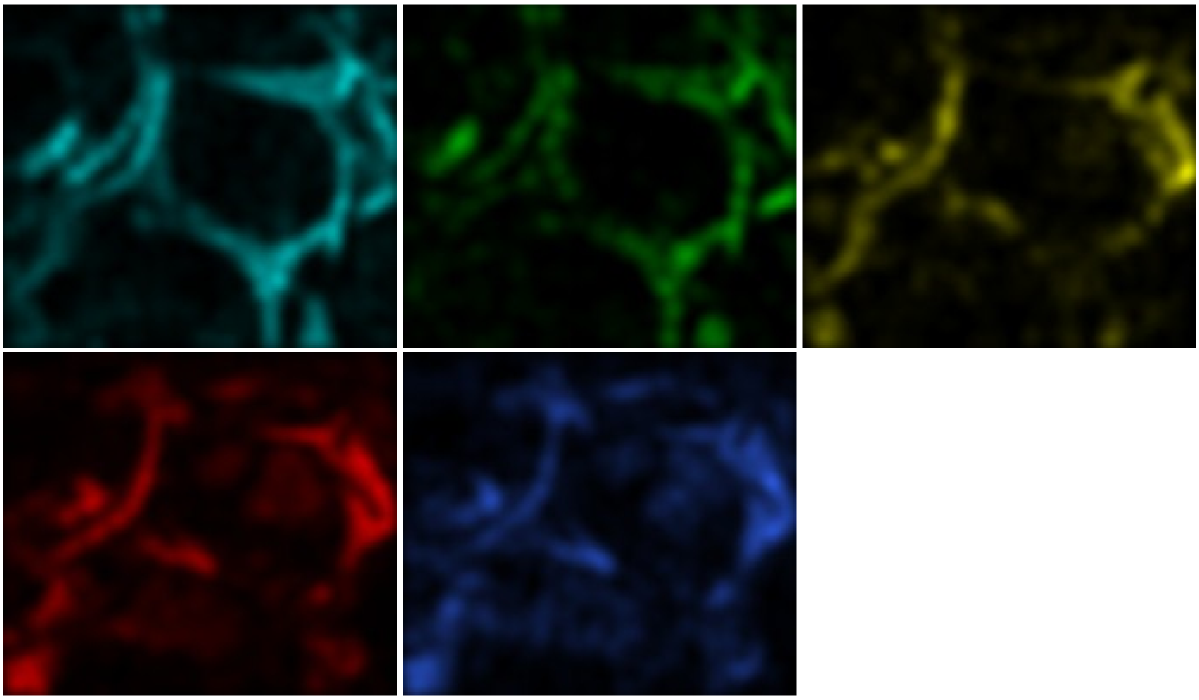

Turquoise = carbon; green = nitrogen; yellow = oxygen; red = silicon; blue = sulfur

Figure S6. Angle-dependent variation of pore size gradient.

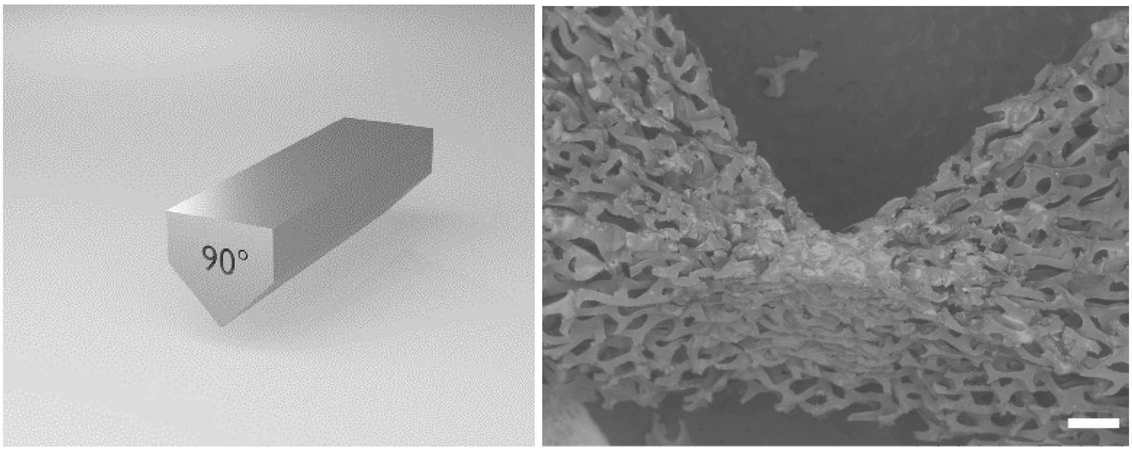

Stamp with an angle of 90°. SEM picture of resulting PU-UKON-2j material. Scale bar 500 µm.

The angle of the kink in the stamp defines the steepness of the gradient in the final material.

Figure S7. Assessment of biocompatibility of organosilica foams before and after biotinylation.

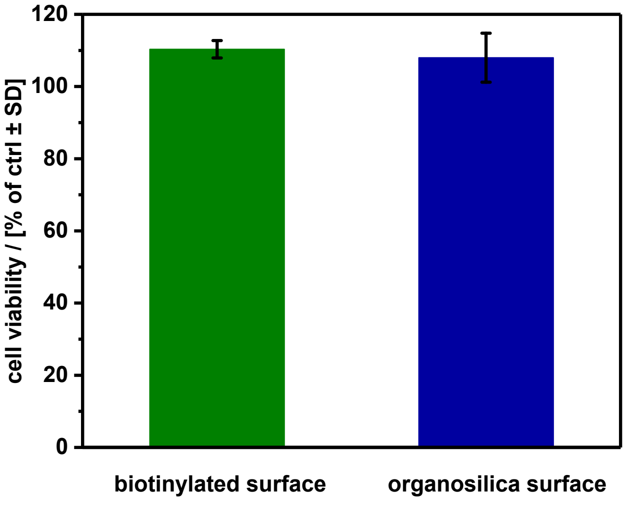

HeLa cells cultured in eluates of biotinylated (green) and unbiotinylated (blue) organosilica foams showed cell growth and viability comparable to control cells grown in pure culture medium.

Cell viability was assessed by lactate dehydrogenase release assay. SD: standard deviation.

Figure S8. Cy5 staining of gradient PU2-UKON-2j materials that have been post-functionalized with biotin.

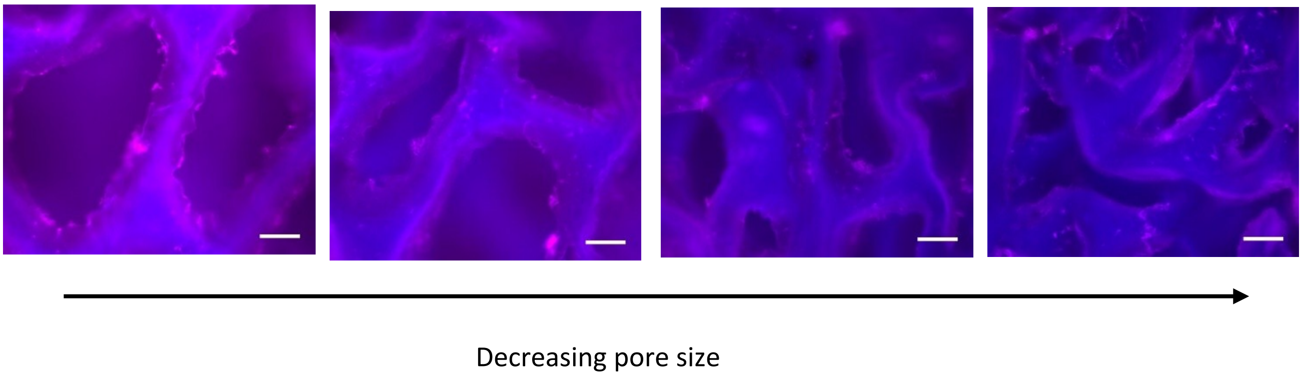

The pictures show Cy5-fluorescence (magenta) and autofluorescence (blue) of thiophenol organosilica foam incubated with Cy5-tagged streptavidin after biofunctionalization of the surface. Pore size decreases from left to right. Scale bar = 100 µm.

The biotinylation of the surface was successful shown by Cy5 fluorescence all over the organosilica foam.

Part B - Chemical experimental details

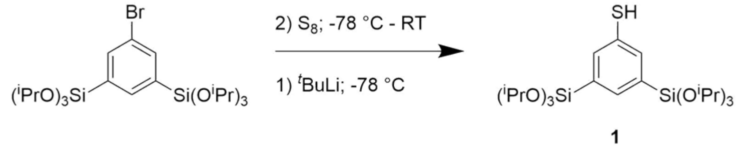

1,5-bistri(*iso*propoxysilyl)-benzene-3-thiol (1, UKON-2j) was synthesized according to a previously reported process[1]. To a solution of 10.6 mmol (6 g, 1 eq.) of 1,3-bistri(*iso*propoxy)silyl-5-bromobenzene in 400 mL of dry diethyl ether was added 21.2 mmol (11.5 mL, 1.9 M, 2 eq.) of *tert*-butyllithium dropwise at -78 °C. The mixture was stirred for 30 min. Then 10.6 mmol (355 mg, 1 eq.) of sulfur was added and stirred for another 30 min at – 78 °C. Afterwards the colorless solution was warmed to room temperature and stirred for 1.5 h. Then the reaction was hydrolyzed with 120 mL of dry *iso*-propanol. After removal of the solvent a yellow oil can be obtained. For further purification column chromatography was applied (silica gel 60, dichloromethane). Finally, 5.38 g (10.37 mmol; >95 %) of a colorless oil was obtained. ^{1}H-NMR (400 MHz, $CDCl_3$): δ (ppm) = 1.20 (d, 36H), 3.42 (s, 1H), 4.24 (sept, 6H), 7.62 (s, 2H), 7.76 (s, 1H). ^{13}C-NMR (400 MHz, $CDCl_3$): δ (ppm) 25.66, 65.52, 129.74, 133.36, 137.50, 138.76. ^{29}Si-NMR (400 MHz, $CDCl_3$): δ (ppm) -62.71. ESI-MS (m/z): calcd for $C_{24}H_{46}O_6SSi_2$ 541.2453 [M+Na$^+$] found: 541.2456.

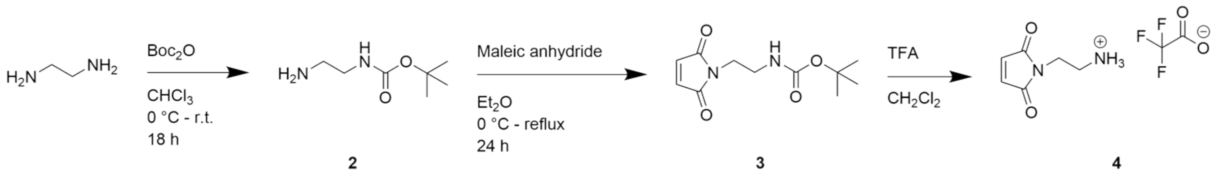

***N*-(*tert*-butoxycarbonyl)-ethyl-1,2-diamine (2)** was synthesized according to a previously reported process[2]. 400 mmol (27 mL, 1 eq.) of 1,2-diaminoethane were dissolved in 400 mL of chloroform at 0 °C. 40 mmol (8.7 g, 0.1 eq.) of di-*tert*-butyl dicarbonate were dissolved in 300 mL of chloroform and added dropwise over a period of 5 h. The reaction was stirred overnight and heated up to room temperature. The solvent was concentrated, and the residue dissolved in 1.5 M sodium carbonate (300 mL) solution. The mixture was extracted with chloroform (3 x 200 mL), the organic phase dried over magnesium sulfate and evaporated to produce a colorless oil. ^{1}H-NMR (400 MHz, $CDCl_3$): δ (ppm) = 1.43 (s, 9H), 2.78 (t, 2H), 3.14 (q, 2H), 4.92 (s, 1H).

1-(*N*-tert-Butoxycarbonyl)-2-aminoethyl) maleimide (3) was synthesized according to a previously reported process[2]. 31.9 mmol (5.1 g, 1 eq.) of *N*-(*tert*-butoxycarbonyl)-ethyl-1,2-diamine (2) and 6.6 mL (47.8 mmol, 1.5 eq.) of triethylamine were dissolved in 60 mL diethyl ether at 0 °C. 31.9 mmol (3.1 g, 1 eq.) of maleic anhydride in 60 mL of diethyl ether were added dropwise to the solution and stirred for 4 h during which the reaction was allowed to reach room temperature. The solvent was evaporated, and the residue was dissolved in acetone (150 mL). 63.7 mmol (8.8 mL, 2 eq.) of triethylamine were added and the reaction was heated to reflux. 47.8 mmol (4.5 mL, 1.5 eq.) of acetic anhydride were added and the reaction was heated to reflux for 20 h. The solvent was removed, the crude product purified via silica column chromatography (ethyl acetate/ pentane 1:1) and resulted in a white solid. ^{1}H-NMR (400 MHz, $CDCl_3$): δ (ppm) = 1.40 (s, 9H), 3.32 (q, 2H), 3.66 (q, 2H), 4.71 (s, 1H), 6.71 (s, 2H).

***N*-(2-Aminoethyl)maleimide trifluoroacetic acid salt (4)** was synthesized according to a previously reported process[2]. 19.2 mmol (4.6 g, 1 eq.) of 1-(*N*-*tert*-butoxycarbonyl)-2-aminoethyl maleimide (3) were dissolved in 35 mL of dichloromethane at 0 °C. 27 mL of trifluoroacetic acid were added to the solution and stirred for 1 h during which the solution was allowed to reach room temperature. The mixture was concentrated, washed with cold

diethyl ether and provided a white solid. ^{1}H-NMR (400 MHz, DMSO-d$_6$): δ (ppm) = 2.99 (t, 2H), 3.66 (t, 2H), 7.06 (s, 2H), 7.93 (s, 3H).

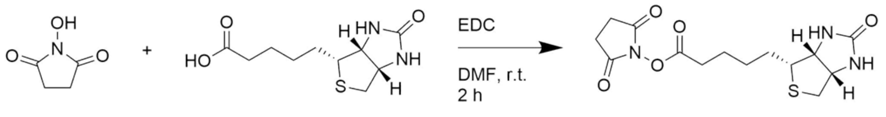

5

Biotin-NHS-ester (5) was synthesized according to a previously reported process[3]. 8.19 mmol (2.0 g, 1 eq.) of biotin were suspended in dry *N,N*-dimethylformamide under inert gas atmosphere. 9.82 mmol (1.13 g, 1.2 eq.) of *N*-hydroxysuccinimide and 9.82 mmol (1.88 g, 1.2 eq.) of *N*-ethyl-*N'*-(3-dimethylaminopropyl)carbodiimide hydrochloride were added and the reaction was stirred for 2 h at room temperature. The solvent was evaporated and the remaining solid washed with a mixture of water/ ethyl acetate and ethanol (95/1/4). ^{1}H-NMR (400 MHz, DMSO-d$_6$): δ (ppm) = 1.36 - 1.71 (m, 6H), 2.59 (m, 1H), 2.68 (t, 2H), 2.82 (s, 2H), 2.86 (m, 1H), 3.12 (m, 1H), 4.16 (m, 1H), 4.31 (m, 1H), 6.36 (s, 1H), 6.42 (s, 1H).

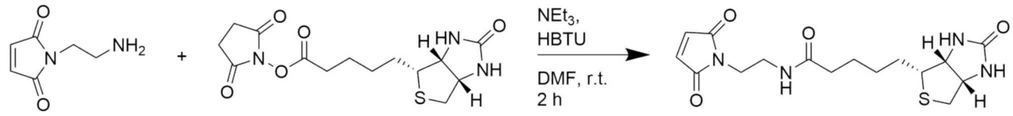

6

Biotin-Maleimide (6, MalBiotin) was synthesized according to a previously reported process[4]. 2.1 mmol (0.75 g, 1 eq.) of Biotin-NHS-ester (5) and 2.8 mmol (0.7 g, 1.3 eq.) of *N*-(2-aminoethyl)maleimide trifluoroacetic acid salt (4) were dissolved in 15 mL of *N,N*-dimethylformamide. 3.6 mmol (0.5 mL, 1.7 eq.) of triethylamine were added, and the reaction was stirred for 3 d at room temperature. The solvent was removed and the solid purified via reverse phase column chromatography (water/ acetonitrile 90/10 → 0/100). ^{1}H-NMR (400 MHz, DMSO-d$_6$): δ (ppm) = 1.19 – 1.68 (m, 6H), 1.95 (t, 2H), 2.57 (m, 2H), 2.83 (s, 2H), 3.09 (m, 1H), 3.18 (q, 1H), 3.44 (t, 1H), 4.13 (m, 1H), 4.30 (m, 1H), 6.34 (s, 1H), 6.40 (s, 1H), 7.00 (s, 2H), 7.85 (t, 1H). ESI-MS (m/z): calcd for $C_{16}H_{22}N_4O_4S$ 389.1262 [M+Na$^+$] found: 389.125.

Ellman test was carried out according to literature. 3 mL of a 20 µM solution of 5,5'-dithiobis-(2-nitrobenzoic acid) (DTNB) in 0.1 M sodium phosphate buffer (pH = 8) were added to a UKON-2j foam. After an incubation time of 2 h the supernatant was analysed by UV/Vis measurement.

Biological experimental details

Cytotoxicity assessment. HeLa cells were seeded in 96 well plates at a density of 60.000 cells/cm^2. After 24 h, medium was exchanged, and cells were cultured in HeLa culture medium that was used to wash the organosilica foams before. After 48 h in wash medium, cytotoxicity was assessed by lactate dehydrogenase (LDH) release assay.

Lactate dehydrogenase (LDH) release assay. LDH activity was measured separately in the supernatant and cell lysate. Supernatant was collected and cells were lysed using 0.5 % Triton X-100 in PBS for 1 h. The percentage of released LDH was calculated as $100xLDH_{supernatant}/LDH_{supernatant+lysate}$. For the enzymatic assay, 10 µl of sample was added to 100 µl of reaction buffer containing NADH (100 µM) and sodium pyruvate (600 µM) in sodium phosphate buffer (pH 7.4, adjusted by 40.24 mM K_2HPO_4 and 9.7 mM KH_2PO_4 buffer). Absorption was measured at 340 nm at 37 °C in intervals of 1 min over a period of 20 min. Enzyme activity was calculated from the slope.

Notes and references

1. J. Gehring, D. Schleheck, B. Trepka, S. Polarz, *ACS Appl. Mater. Interfaces* **2015,** *7* (1), 1021-1029.
2. M. Richter, A. Chakrabarti, I. R. Ruttekolk, B. Wiesner, M. Beyermann, R. Brock, J. Rademann, *Chem. Eur. J.* **2012,** *18* (52), 16708-16715.
3. Y. Kimura, S. Ito, Y. Shimizu, M. Kanai, *Org. Lett.* **2013,** *15* (16), 4130-4133.
4. H. Shi, K. Liu, A. Xu, S. Q. Yao, *Chem. Commun.* **2009,** (33), 5030-5032.
